# From pollinosis to digestive allergy

**DOI:** 10.1186/2045-7022-3-S3-P31

**Published:** 2013-07-25

**Authors:** L Neziri-Ahmetaj, A Neziri, K Fatime

**Affiliations:** 1 Allergology and Immunology, O.Sh.Ylli, Prishtina, Kosovo

## Background

Pollinosios is defined as a appearance of respiratory symptoms(rhinoconjuctivitis and/or asthma) as a result of the inhalation of pollen to which the individual is sensitized. Pollen can act as a source of allergens that induce primary senzitisation in the host as a result of inhalation,with secondary allergy to plant foods containing shared allergens via cross-reactivity mechanisms. On the other hand,in a study including individuals sesitised to ARTEMISIA (mugwort-in compositae family),food sensitivity rate was found to be 23.7% and 60% of these cases who were sensitive to food and Artemisia pollen, were also sensitive to honey. Honey is produced by domesticated and many wild bees from flower nectar and other plant secretions.over 180 different compound and 22 sugars have been found in honey.honey contains many volative components,which at least 35 have been identified.Honey also contains choline and acetyl choline.

## Methods

A case report. Even a few adult cases have been reported about anaphylaxis occurred by honey,we can introduce a 52 years old women Shaqiri H, with Angioedema acuta. She presented anaphylaxis a few years ago after honey ingestion,developed within 10 minutes with urticaria, angio-oedema, cough and wheezing. She came in our clinic (allergy center, Ylli) for further evaluation,with diagniosis Angioedema acuta.

## Results

The skin prick test in inhalants presented positivity in mugwort (15/40mm). Also skin prick test in nutritive allergens presented positivity to chamomile (5/15mm). We could not perform oral challenge to confirm the diagnosis considering the life threating food reaction history.

## Conclusion

Even though anaphylaxis by honey is rare,we cannot forget that food allergies could be life threatening, among them very rare but life threatening can be honey, in patients’ sensitization from different kind of pollens.

## Disclosure of interest

None declared.

